# NSAIDs and Cell Proliferation in Colorectal Cancer

**DOI:** 10.3390/ph3072007

**Published:** 2010-06-24

**Authors:** Raj Ettarh, Anthony Cullen, Alvise Calamai

**Affiliations:** School of Medicine & Medical Science, University College Dublin, C206 Health Sciences Building, Belfield, Dublin 4, Ireland; E-Mails: Anthony.Cullen1@ucdconnect.ie (A.C.); alvise.calamai@gmail.com (A.C.)

**Keywords:** colon cancer, cyclooxygenase, COX inhibitors

## Abstract

Colon cancer is common worldwide and accounts for significant morbidity and mortality in patients. Fortunately, epidemiological studies have demonstrated that continuous therapy with NSAIDs offers real promise of chemoprevention and adjunct therapy for colon cancer patients. Tumour growth is the result of complex regulation that determines the balance between cell proliferation and cell death. How NSAIDs affect this balance is important for understanding and improving treatment strategies and drug effectiveness. NSAIDs inhibit proliferation and impair the growth of colon cancer cell lines when tested in culture *in vitro* and many NSAIDs also prevent tumorigenesis and reduce tumour growth in animal models and in patients, but the relationship to inhibition of tumour cell proliferation is less convincing, principally due to gaps in the available data. High concentrations of NSAIDs are required *in vitro* to achieve cancer cell inhibition and growth retardation at varying time-points following treatment. However, the results from studies with colon cancer cell xenografts are promising and, together with better comparative data on anti-proliferative NSAID concentrations and doses (for *in vitro* and *in vivo* administration), could provide more information to improve our understanding of the relationships between these agents, dose and dosing regimen, and cellular environment.

## 1. Introduction

Colon cancer is common worldwide. Nearly a million people develop the disease every year and in the United States alone, there are 50,000 cancer-related deaths annually [1]. The mechanisms that lead to and sustain carcinogenesis have been investigated extensively and are multifactorial. Irrespective of the initiating or sustaining causes, however, the primary manifestation of cancer is excessive growth of cells and tissue. Growth of normal and abnormal tissue is achieved by a balance between cell production and cell death *i.e.* proliferation that is counterbalanced by apoptosis and necrosis [2]. 

There is a wealth of experimental evidence demonstrating that colon cancer is associated with dysregulation and overexpression of the prostaglandin-synthesizing enzyme cyclooxygenase (COX) as well as accompanying overproduction of prostaglandin, abnormal cell and tissue adjustments in relation to vascularization, cell adhesion, apoptosis and proliferation. One important theme in the body of evidence is the fact that non-steroidal anti-inflammatory drugs (NSAIDs) are able to alter intestinal tumour growth rates and modulate carcinogenesis by a variety of reported methods including inhibition of COX activity and disruption of prostaglandin homeostasis, interruption of nuclear factor kappa B (NF-kB) signaling [3,4,5,6], and of extracellular signal-regulated kinases (ERK/MAPK) [7], induction of various apoptotic pathways [8,9,10], as well as effects on cell cycling [11,12,13,14]. All of these mechanisms either contribute and enhance, or antagonise and counterbalance, the proliferative behaviour that is observed in tumour cells.

This review will focus on assessment and evaluation of the data that is currently available on the effects on cell proliferation in the enteric epithelium from pharmacological intervention with NSAIDs.

## 2. Wnt and Colon Cancer

A majority of NSAIDs inhibit the cyclooxygenase enzymes (COX-1 and COX-2) and in the process interrupt the biosynthetic conversion of arachidonic acid to eicosanoids [15]. COX-1 is expressed widely in tissues, including the gastric mucosa and platelets, while the normally low level of COX-2 is rapidly increased via induction by pro-inflammatory cytokines [15,16]. Increased levels of expression of COX-2 are associated with colorectal tumours: up to 85% of colorectal adenomas and carcinomas express COX-2 [17,18,19,20]. 

The Wnt pathway plays an important role in the regulation of cell proliferation and differentiation. Disruption of this carefully balanced regulation leads to disordered proliferation. Wnt signalling helps to control the levels of cellular beta catenin, between pools bound to adenomatous polyposis coli (APC) and to the cell adhesion molecule E-cadherin. Wnt ligand signalling leads to interaction with membrane receptor proteins (frizzled proteins) that are receptors for Wnt ligands. The interaction triggers a signalling cascade that alters the relationship between the scaffold protein Axin and glycogen synthase kinase-3beta (GSK3beta). Axin binds adenomatous polyposis coli APC to the multidocking protein beta-catenin. GSK3beta phosphorylates beta catenin for ubiquitin-mediated proteosomal degradation [21,22].

Wnt-induced alteration of Axin interaction leads to release of beta catenin from the APC-GSK complex so that unphosphorylated beta catenin is stabilised in the cytoplasm and able to translocate to the nucleus [23,24,25,26,27,28] where it associates with transcription factors of the TCF/Lef family and other co-factors to form complexes that activate downstream target genes that regulate proliferation, differentiation and apoptosis: c-myc, cyclin D1 and COX-2 [29,30,31,32].

As with many cancers, colorectal cancers develop from accumulated mutations, deletions or truncations in oncogenes and tumour suppressor genes such as APC, ras and p53 [33]. The loss of functional APC is associated with colon cancer and this is evident in mice with mutant APC: these APCmin mice develop multiple intestinal tumours [34]. Similar mutations in APC in humans results in the hereditary condition familial adenomatous polyposis (FAP) in which multiple colonic polyps develop in patients, with a potential for these polyps to progress and become adenomatous or carcinomatous [35]. These polyps show significant expression of COX-2 and therefore represent a target for therapeutic control with NSAIDs [36,37].

## 3. NSAIDs Inhibit Colon Cancer Cell Proliferation *in vitro*

A variety of colorectal tumour cell lines have been used in *in vitro* investigations of the effects of NSAIDs on several aspects of tumour initiation and progression (Table 1).

When HT29 colorectal adenoma cell lines are treated with different NSAIDs, proliferation is reduced as early as 48 hours after treatment with naproxen and piroxicam. Similar anti-proliferative effects have been reported with indomethacin after 72 hours [38], aspirin after 96 hours [38], NS398 after 72 hours [39], 5-aminosalicylic acid after 48 hours [40], and with sulindac sulfide after 24 hours [41]. However, Piazza and colleagues [42] were unable to inhibit growth in these same cell lines with aspirin, naproxen or salicylic acid [42], but found that ibuprofen and sulindac sulfoxide inhibited growth with a low potency in contrast to higher potency shown with indomethacin, sulindac sulfide and diclofenac. They also suggested that their data (for these compounds) showed no relationship between potencies for inhibition of COX, and effects on growth or apoptosis.

With HCA-7 colorectal carcinoma cells, reduced proliferation was evident following treatment with celecoxib within 12 hours [43]. In RKO rectal cancer cell lines proliferation was inhibited after 72 hours following exposure to NS398 and sulindac [39] and with HT115 colon cancer cell lines, anti-proliferative effects were reported within 24 hours following treatment with 5-aminosalicylic acid. Using SW620 cell lines, the inhibition of proliferation by indomethacin and sulindac is apparent between 24–72 hours after administration of these agents to culture [44,45]. All of these colon cancer cell lines express COX.

When cell lines that do not express COX are treated with NSAIDs, proliferation is also inhibited. Celecoxib reduces proliferation in HCT116 colon cancer cells after 72 hours [46] and the anti-proliferative effect seen in these cells with indomethacin and nimesulide occurs earlier [47]. Similarly in HT15 colon cancer cell lines, treatment with celecoxib, sulindac and piroxicam inhibits proliferation at various timepoint between 24–72 hours and in DLD-1 colon cancer cell lines, proliferation is reduced after 24 hours when treatment with 5-aminosalicylic acid is applied [48]. These reports suggest that the anti-proliferative effect of these NSAIDs may not be related to their ability to inhibit cyclooxygenase and that non-COX mechanisms might be involved. This is reflected in the extensive data that has been generated from studies that have investigated other pathways and mechanisms: NF-kB, ERK/MAPK, peroxisome proliferator-activated receptor gamma (PPARγ), caspases, and cyclins.

Most of the evidence allows the conclusion that NSAIDs inhibit proliferation of colon cancer cells *in vitro* and that the mechanism of action for the anti-proliferative activity shown by NSAIDs appears to be unrelated to cellular COX expression and activity.

**Table 1:** Colon cancer cell lines and the concentration of NSAIDs used in studies that show growth inhibition. The time-point at which inhibition of cell growth is recorded is also presented. Non-COX-expressing cell lines are presented in the lower part of the table. Studies are identified according to the numerical listing given in the bibliography. ^1^ number of viable/attached cells, ^2^ MTT assay, ^3^ CFSE labeling, flow-cytometry, ^4^ growth ratio: number of treated/untreated cells. HT29, HCA7, HT115 and SW620 express COX-2; RKO express COX-1 and COX-2. 5-ASA = 5-aminosalicylic acid, CFSE = carboxyfluorescein diacetate succinimidyl ester, MTT = 3-(4,5-dimethylthiazol-2-yl)-2,5-diphenyltetrazolium bromide.

Cell Line	NSAID	Dose uM	Effect Proliferation	Time-Point	Ref.
**COX-expressing**					
HT-29	Aspirin	400	down^1^	96 h	[26]
HT-29	Indomethacin	100	down^1^	72 h	[26]
HT-29	Naproxen	200	down^1^	48 h	[26]
HT-29	Piroxicam	300	down^1^	48 h	[26]
HT-29	sulindac sulfide	185	down^1^	24 h	[29]
HT29	sulindac sulfide	120	down^1^	24 h	[30]
HT29	sulindac sulfone	150	down^1^	72 h	[27]
HT29	NS398	120	down^1^	72 h	[27]
HT29	5-ASA	36000	down^2^	48 h	[28]
HCA-7	celecoxib	50	down^2^	12 h	[31]
RKO	NS398	120	down^1^	72 h	[27]
RKO	sulindac sulfone	150	down^1^	72 h	[27]
HT115	5-ASA	5000	down^3^	24 h	[36]
SW620	sulindac	1600	down^2^	24–72 h	[32]
SW620	indomethacin	400	down^2^	96 h	[33]
**Non-COX-expressing**					
HCT116	indomethacin	600	down^1^	24–72 h	[35]
HCT116	nimesulide	100	down^1^	24–72 h	[35]
HCT116	celecoxib	12.5	down^4^	72 h	[34]
HT-15	celecoxib	50	down^2^	12 h	[31]
HT-15	sulindac sulfide	200	down^1^	24 h	[29]
HT-15	piroxicam	900	down^1^	72 h	[29]
DLD-1	5-ASA	5000	down^3^	24 h	[36]

## 4. Anti-Proliferative Dose Range of NSAIDs *in vitro*

Much of the available evidence indicates that most NSAIDs inhibit tumour cell proliferation *in vitro*. However, these effects are influenced by several factors including micromolar drug concentrations, duration of treatment, and timepoint at which anti-proliferative effects are assayed. Sulindac sulfone, a sulindac metabolite that does not have anti-inflammatory properties, is anti-proliferative in HT29 COX-expressing colon cancer cell lines within 72 hours when delivered at a concentration of 150 μM, whereas the active anti-inflammatory sulfide metabolite of sulindac inhibits proliferation within 24 hours when administered at a concentration of 120–185 μM [39,41,42]. Other studies in SW620 colon cancer cell lines have employed concentrations of sulindac of up to 1,600 μM to inhibit proliferation in 24–72 hours [44]. Concentrations of between 100–400 μM are required to inhibit proliferation in COX-expressing colon cancer cell lines using aspirin, indomethacin, naproxen and piroxicam [38,45]. 

In tumour cell lines that do not express COX, a variety of concentrations of NSAIDs have been shown to inhibit proliferation—ranging from 100 μM of nimesulide to 600 μM of indomethacin in HCT116 colon cancer cell lines [47]. Interestingly, concentrations as low as 12.5–50 μM of celecoxib have been employed to reduce proliferation in these cells [43,46]. Many of these studies have reported the effects of various concentrations of NSAIDs on proliferation in tumour cell lines but few have investigated these effects simultaneously in COX-expressing and non-COX-expressing cell lines while controlling for drug concentration and time. For instance, 300 μM of piroxicam inhibits proliferation in HT29 COX-expressing colon cancer cells after 48 hours, but 900 μM of the drug is anti-proliferative in non-COX-expressing HT-15 colon cancer cells after 72 hours [38,41]. Two separate studies using indomethacin in HT29 and non-COX-expressing HCT116 colon cancer cell lines demonstrated anti-proliferative effects with concentrations of 100 μM and 600 μM of the drug, respectively, after 72 hours [38,47]. It is difficult to draw meaningful conclusions about the relative contribution of the individual influences of COX, concentration and time from the data in these studies. Stolfi and co-workers [48] have recently shown that a high concentration (5,000 μM) of the drug mesalazine (5-aminosalicylic acid) inhibits proliferation after 24 hours in COX-expressing HT115 and DLD-1 non-COX-expressing colon cancer cell lines. Further carefully designed investigations are required to provide data on the individual and collective influences of COX, *in vitro* drug concentrations and duration of treatment for various NSAIDs. 

Given the wide range of doses of the different NSAIDs that have been used and shown to inhibit proliferation, it is reasonable to conclude that for any given NSAID, it is possible to find an appropriate concentration that will inhibit proliferation if a wide range of concentrations is tested.

## 5. NSAIDs Inhibit Colon Cancer Cell Proliferation *in vivo*

Most of the available data on the effect of NSAIDs on colonic tumour proliferation *in vivo* relate to studies carried out using animal models of colonic tumours (Table 2). Colonic tumours have been induced chemically using *N*-methyl-*N*-nitrosourea, 1,2-dimethylhydrazine (DMH) or azoxymethane (AOM). One common genetic model that has been employed is the APC min mouse because it is a closely mimics human familial adenomatous polyposis (FAP), a condition in which multiple polyps develop in the colon. These APC min mice also develop numerous polyps in the small intestine and this allows for investigations that enumerate and compare polyps between treatment regimes. The administration of various NSAIDs in these animal models has been shown to be associated with inhibition of initiation and progression of polyps as well as a reduction in the tumour load. Mahmoud and colleagues [49] showed that longterm administration of sulindac sulfide in drinking water (11 weeks) reduced the development of small intestinal polyps in APC min mice, an effect that was absent in the colon [49,50]. However in DMH-treated mice, although development of colonic tumours was prevented by treatment with sulindac for 24 weeks, colonic cell proliferation increased after 18 weeks of sulindac treatment [51,52]. Similar NSAID-induced chemoprevention in animal models has been reported with indomethacin [53], aspirin [54], piroxicam [55], celecoxib [56,57], and nimesulide [58]. All of these studies are characterized by the administration of NSAIDs over a lengthy treatment period ranging from 6–46 weeks. 

**Table 2:** The doses of NSAIDs administered *in vivo* in tumour model experiments and their effect on initiation and progression of carcinogenesis. The duration of treatment is presented and the effect on proliferation is indicated. NMNU = n-methyl-N-nitrosourea, AOM = azoxymethane, DMH = 1,2-dimethylhydrazine, APC = adenomatous polyposis coli, NA = not measured.

NSAID	Dose ppm	Duration	Proliferation	Inhibition Effect	Model	Rodent	Ref.
**Small Intestine**							
celecoxib	1,500 ppm	6 wks	NA	tumor number	APC	C57BL	[44]
piroxicam	50–200 ppm	6 wks	NA	tumor number	APC	C57BL	[43]
sulindac sulfide	20 mg/kg	11 wks	NA	tumorigenesis	APC	C57BL	[37]
**Colon**							
indomethacin	10 ppm	1–30 wks	NA	tumorigenesis	NMNU	Fisher rats	[41]
aspirin	200–400 ppm	52 wks	NA	tumorigenesis	AOM	F344 rats	[42]
nimesulide	0.04% w/w	14 wks	down	NA	AOM	CD-1 mice	[46]
celecoxib	300 ppm	46 wks	down	NA	AOM	F344 rats	[45]
celecoxib	1,500 ppm	6 wks	NA	tumor number	APC	C57BL	[44]
sulindac	5 mg/kg	18 wks	up	NA	DMH	BALB/C	[40]
sulindac	5 mg/kg	24 wks	NA	tumorigenesis	DMH	BALB/C	[39]
sulindac	160 ppm	10 wks	NA	no change	APC	C57BL	[38]
sulindac sulfide	20 mg/kg	11wks	NA	no change	APC	C57BL	[37]

Prevention of tumorigenesis or reduction in tumor load can be a reflection of decreased cell proliferation or of increased cell death. For many of these studies, the data on cell proliferation or cell death was either not sought or else not presented. Whereas the data for NSAID effects on cell proliferation from *in vitro* studies is unequivocal in their demonstration of anti-proliferation, the limited data on cell proliferation from *in vivo* studies using animal models of colon cancer shows more conflicting findings. Indomethacin, aspirin and piroxicam have beneficial effects in tumorigenesis and reduction in colonic tumour load *in vivo* but whether these effects are related to anti-proliferative action by the NSAIDs is not certain [53,54,55]. Celecoxib treatment reduces tumor numbers and inhibits cell proliferation [56,57]. The data on the various forms of sulindac suggests that the effect of this drug on tumorigenesis is variable and may be dependent on the animal model employed [49,50,51,52]. 

Some of this inconsistency in the findings from studies in animal models is reflected in the results of investigations in patients. In a study of patients with FAP, treatment with sulindac (150 mg twice daily) for nine months reduced the number and size of colorectal adenomas [59]. A second study found that treatment with standard doses of sulindac (25–150 mg twice daily) for 48 months did not prevent adenomas in patients [60]. However several clinical trials have shown beneficial prevention of colorectal cancer in patients following treatments with aspirin and celecoxib [61,62,63]. 

## 6. Anti-Proliferative Dose Range of NSAIDs *in vivo*

Many of the studies in animal models have used doses of NSAIDs that would not ordinarily be employed clinically in patients. There are however very few studies that have used clinically relevant doses of NSAIDs to investigate colon cancer in animal models. Moorghen and co-workers [52] examined cell proliferation in the colonic mucosal epithelium in mice following treatment with low-dose sulindac for 18 weeks. Their finding of sulindac-mediated increase in cell proliferation led them to question the rationale behind the therapeutic manipulation of crypt cell proliferation in order to reduce the risk of colon cancer. 

While the main focus has been on investigating the treatment of colon cancer with NSAIDs, very little attention has been given to the effect on or the response of the normal bowel following NSAID treatment (Table 3). For example, what happens to proliferation in the unaffected (non-adenomatous) parts of intestine in colon cancer models following treatment with NSAIDs? The distinction between affected and unaffected parts of the bowel is important because COX-2 is upregulated in 90% of human colon cancer and 40% of premalignant adenomas of the colon but is absent in the normal colon [64,65]. This provides some of the rationale for *in vitro* studies that employ non-COX-expressing colon cancer cell lines. Hollingshead and colleagues [47] found that proliferation was inhibited in HCT116 non-COX-expressing colon cancer cells and in normal colonic epithelium in mice when treated with different *in vitro* and *in vivo* doses of nimesulide for 72 hours and five days respectively. With a large dose of celecoxib administered over 45 days, Williams and co-workers [43] found that mitosis remained normal in the small and large bowel. In the same study, they found that growth of HCA-7 colon cancer cell xenografts reduced with an identical treatment regime with celecoxib. Similar growth inhibition of xenografted colon cancer cells has been observed with the sulindac derivative SS amide [66]. 

When CD-1 mice were treated twice daily with clinically relevant doses of nimesulide or indomethacin and examined after six and 24 hours, changes in small intestinal cell proliferation were found to be dependent on enteric region and duration of treatment [67,68]. Indomethacin down-regulated cell production in proximal intestinal crypts after six hours while nimesulide reduced cell proliferation in proximal and distal intestinal crypts. After 24 hours of treatment with either drug, the numbers of proliferating cells in the crypts showed regional variation with increases in some regions and reductions in others. The number of cells produced by these crypts however was downregulated in indomethacin-treated mice but remained, in nimesulide-treated mice, similar to the numbers in untreated mice.

**Table 3:** NSAIDs and enteric cell proliferation *in vivo*. The duration of treatment is indicated.

NSAID	Dose	Duration	Proliferation	Model	Ref.
**Small Intestine**					
celecoxib	1,250 mg/kg chow	45 days	No change	C57BL	[31]
indomethacin	10 mg/kg body weight	6 h	variable	CD-1	[55]
indomethacin	10 mg/kg body weight	24 h	variable	CD-1	[56]
nimesulide	10 mg/kg body weight	6 h	down	CD-1	[55]
nimesulide	10 mg/kg body weight	24 h	variable	CD-1	[56]
**Colon**					
celecoxib	1,250 mg/kg chow	45 days	No change	C57BL	[31]
nimesulide	400 mg/kg	5 days	down	C57BL	[35]

Duration of treatment with NSAIDs is also important. While it is recognized that there will be different regimes (dose, duration) for each NSAID, drawing conclusions from the available evidence is impaired by the variety in application of treatment regimes in these studies. There may be a need to begin to differentiate between short term (acute) and longterm (adaptive) treatment regimes with respect to the anti-proliferative effects of NSAIDs in the colon. Most of the *in vitro* studies have reported effects for NSAIDs over a time period of between 24–96 hours. There is a need for correspondingly short *in vivo* investigations with NSAIDs in order to allow comparisons of the data and a better understanding of the relationships between drug, cell environment and measured endpoint. There is also a need to explore ways of extending the duration of *in vitro* studies with colon cancer cell lines in order to narrow the gap between the *in vitro* study design and *in vivo* treatment regimes.

## 7. NSAIDs and Other Cancers

While the data reviewed so far has been obtained from studies examining influences on colorectal cancer, there is extensive literature on the relationship between various NSAIDs and other cancers in the skin, stomach, breast, prostate, pancreas, ovary and the urinary bladder (extensively reviewed by Baron) [69]. While the findings from these have contributed to our understanding of the complexity of the relationship between NSAIDs and tumours, data evaluations from population-based studies continue to provide conflicting conclusions. 

Some epidemiological studies suggest that the use of aspirin do not reduce the risk of PSA-detected prostate cancer while other investigations have found that aspirin may be useful in populations with a high risk of developing prostate cancer [70,71]. The relationship between NSAIDs and prostate cancer also appears to depend on prior, regular or current usage of these drugs [72,73]. Conflicting outcomes have also been reported from studies of breast cancer risk and usage of various NSAIDs [74,75] and led Cuzick *et al.* [76] to conclude that, despite the wealth of published reports, there is still insufficient data on risk-benefit profiling of NSAIDs for use in cancer chemoprevention. They suggest that more controlled trials with new study designs are necessary.

With these non-colorectal cancers, the mechanisms by which NSAIDs affect tumour behaviour are also variable and include disruptions of signalling pathways as well as interruptions of metabolism of other therapeutic agents (if used in combination treatments) [77].

## 8. Conclusions

The available evidence suggest the followings:

1. At an appropriate concentration *in vitro*, most NSAIDs inhibit proliferation in colon cancer cell lines.
2. When administered *in vitro* to colon cancer cell lines in culture, the limited evidence available indicates that clinically realistic doses of NSAIDs do not inhibit cell proliferation. There is evidence demonstrating that clinical doses of NSAIDs inhibit proliferation and reduce tumour growth in xenografted tumours derived from colon cancer cell lines.
3. Acute effects on proliferation seen *in vitro* with NSAIDs treatment cannot be tested/reproduced following treatment *in vivo* because the concentrations used *in vitro* are toxic and lethal to cells, tissue and organs *in vivo*.
4. Many NSAIDs will prevent carcinogenesis and slow tumour growth in animal models but the limited data relating to the ability of NSAIDs to inhibit tumour proliferation is mixed.
5. The concentrations of NSAIDs required to inhibit colorectal cancer cell proliferation *in vitro* are much higher than the doses required to inhibit cell proliferation in colonic tumours *in vivo*.
6. In studies with colon cancer cell lines, it has proven difficult to reproduce the adaptive effects on proliferation seen *in vivo* following NSAIDs administration, due largely to methodological and technical limitations imposed by a cell culture approach.
7. A distinction between acute effects (within the first 96 hours) and adaptive effects (long term treatment) that are observed when treatment is given over weeks and months may be necessary in order to allow more useful and appropriate comparisons between data sets.
8. Better comparative data is required from *in vitro* and *in vivo* studies using low doses of NSAIDs over short and long durations of treatment, and from studies of NSAID effects in normal colonic tissue.

The published evidence from clinical studies and patient therapy indicates that aspirin, sulindac and celecoxib offer promise for effective use in colon cancer. Further studies are needed to establish useful *in vitro* and *in vivo* rodent models that reproduce the clinical data in terms of dosages, duration of treatment and effect on tumour tissue and cells. These models might then provide the basis for targeted experiments and investigations into mechanisms of actions of the NSAIDs used.
